# The influence of habitual consumption of chewing gums in the outcome of masticatory performance tests using two-coloured chewing gums

**DOI:** 10.1038/s41598-019-42918-z

**Published:** 2019-04-25

**Authors:** Gustavo Vaccaro, José Ignacio Peláez, José Antonio Gil-Montoya

**Affiliations:** 1 International Postgraduate School, School of Dentistry, Granada University, Granada, Spain; 2 Biomedical Research Institute of Malaga (IBIMA), Málaga, Spain; 3 Department of Languages and Computer Sciences, University of Malaga, Malaga, Spain; 4 Prometeo Project, National Secretary of Higher Education, Science, Technology and Innovation (SENESCYT), University of Guayaquil, Guayaquil, Ecuador; 5 Gerodontology Department, School of Dentistry, Granada University, Granada, Spain

**Keywords:** Mandibular muscles, Oral manifestations, Biomedical engineering

## Abstract

The aim of this study is to assess the influence of regular consumption of chewing-gums on the Masticatory Performance (MP); and to determine if increasing the consumption improves the MP of non-regular consumers. We recorded the chewing-gums consumption rate (CGC) and measured the MP of 265 participants (µ = 47.09, σ = 22.49 years) using the Variance of the Histogram of the Hue (VhH) image processing method. Then, participants were instructed to increase the consumption, and the MP was measured again (SESSION) two and four days after. Normality of MP was verified with Kolmogorov-Smirnov and Shapiro-Wilk tests. The association between the age and the consumption rate was measured with GEE and the eta-squared statistic. Finally, a 3 × 3 mixed ANOVA with SESSION as the within-subject factor and CGC as the between-subjects factor was run. Session-wise and group-wise comparison were performed with post hoc Bonferroni. No systematic error was detected for VhH (*p* = 1.00). Kolmogorov-Smirnov and Shapiro-Wilk tests confirmed the normality of the distribution of MP (*p* > 0.05). There was a significant effect of SESSION on MP, *F*(1.746, 457.328) = 59.075, p < 0.001; furthermore, there were significant differences in MP between SESSIONs. Additionally, there was a significant effect of CGC on MP, with *F* (2, 356.53) = 564.73, p < 0.001. In conclusion, the chewing-gum consumption habits influence the two-coloured chewing gum mixing test. The apparent MP of non-regular consumers can be improved by prescribing a controlled increase in the consumption of chewing-gums for a few days.

## Introduction

The Masticatory Performance (MP) is an indicator of oral function capabilities that measures the comminution of food attainable under standardized testing conditions^1^. It is possible to generalize that the MP quantifies the changes of a given characteristic in the food bolus during mastication; for example, the average particle size of hard/brittle food such as peanuts^2^ or Optosil^3^, or the mixture of colours of a chewing gum^4^. The MP is commonly used to assess the impact of prosthetic dental treatments^5–7^; besides, previous studies have associated numerous health disorders with a decline in MP, such as the orofacial impairments following stroke^8,9^, the Metabolic Syndrome^10^, among others^11^. Furthermore, MP assessment can be a valuable tool for geriatric care services, that are often required to evaluate the functional impairments of individuals in faster and more accurate ways while using less invasive methods.

One of the fastest and easiest routines for objective MP assessment is the quantification of the mixture of a two-coloured chewing-gum specimen subjected to mastication^4,12–14^. Several studies proposed digital image analysis approaches for mixture quantification, ranging from simple feature extraction procedures^13,15^, to complex multi-feature comparisons^4,12,16^ and pattern recognition using computational intelligence^17^. The two-coloured chewing-gum mixture quantification procedures are simple and easy to reproduce, as they do not require specialized equipment or training. However, MP measures obtained this way are prone to high standard deviations regardless of the image processing approach^12,17^. Therefore, current research in this area aims to enhance the accuracy of the procedure.

In this regard, we explore the possibility that the regular consumption of chewing-gums increases the chances of achieving a greater mixture degree on two-coloured chewing gum mixing tests. The effects of food preference on MP measures has been suggested in previous studies that used other MP assessment methodologies, such as the analysis of fragmentation of hard and brittle test-foods^18–22^. However, to the best of our knowledge, there are no studies in scientific literature that consider the affinity of participants towards chewing-gums as a modifying factor for the MP. In this regard, a few questions arise: firstly, do regular chewing-gum consumers score better MP values than non-regular consumers? Furthermore, can a non-regular consumer score better MP if instructed to increase the chewing gum consumption rate? Within this context, there is some evidence that the chewing-gum consumption rate may be correlated to the age^23^; therefore, it is important to consider other confounding factors such as number of natural teeth, the usage of dental prothesis, and TMJ disorders.

The aim of this study is to assess the influence of regular consumption of chewing-gums on the MP when using the two-coloured chewing-gum mixing test approach. The following null hypotheses were tested:

- There is no association between chewing-gum consumption rate and the age.
- There are no differences in the mean MP between individuals with different chewing-gum consumption rates.
- Individuals that consume less than one chewing-gum per month will not show differences in their mean MP after being prescribed with increasing the consumption of chewing-gums for two days.

## Materials and Methods

### Participants

Two hundred and sixty-five participants were recruited: 122 females, ranging from 18 to 89 years old (µ = 47.59, σ = 23.03); and 143 males, ranging from 18 to 88 years old (µ = 48.51, σ = 21.68). This experiment considered four age groups (AG): less than 24 years old (AG = 1), between 25 and 44 years old (AG = 2), between 46 and 65 years old (AG = 3), and more than 65 years old (AG = 4).

Subjects were either dentistry students or patients being treated at the Faculty of Dentistry of the University of Guayaquil, Ecuador. The inclusion criteria were: being 18 to 90 years old, having at least 28 natural or prosthetic teeth (including full dentures), a DMFT score of 2 or less for participants with natural teeth, and self-perception of the mastication as normal. Exclusion criteria were hypersensitivity or allergies to any of the ingredients of the test-food, defective or poorly supported dentures, self-perception of not being able to chew on a chewing-gum, orofacial pain, bruxism, tooth wear, TMJ dysfunction symptoms, and the usage of orthodontic appliances. Written informed consent was obtained from all participants. Formal approval through the Ethical Committee for Human and Animal Experimentation of the University of Guayaquil was obtained for this experiment. Furthermore, this study was performed in accordance with relevant guidelines and regulations.

### Experimental design

#### Test-food

This experiment was performed within the scope of a larger study. In this case two flavours of 5™ chewing-gums manufactured by the Wrigley Company were selected: “Celsius” (red dye), and “Electro” (green dye). These are commercially available in Spain in the form individually wrapped strips measuring 1.5 × 20 × 75 mm. A trained operator formed the test-food specimens by manually unwrapping and stacking two pieces of both colours. These chewing-gums were imported to Ecuador for the sole purpose of this experiment. The selection of the test-food for this study followed the specifications presented by Schimmel, *et al*. (2015), such that: specimens must have two different colours, the colours must mix when subjected to mastication, must not have a hard coating, and must not stick to artificial dentures^4^.

This study recorded the age (numerical scalar), sex (nominal: male or female), dental status (DS) (nominal: natural or artificial denture), and the per-month consumption rate of chewing gums (nominal: low, medium, or high); where low consumption rate accounts for less than 1 chewing-gum, medium consumption rate for between 1 and 4 chewing-gums, and high consumption rate for more than 4 chewing gums, per month.

#### Clinical procedure

First, an operator instructed the patient to chew on a test-food sample for 20 chewing strokes, on the preferred mastication side and at a comfortable speed^12,16,17^. Secondly, the masticated bolus was retrieved and placed between two transparent plastic sheets. Thirdly, the wafer composed of the chewing-gum and plastic sheets was pressed to a 1 mm thick using a screw-driven press^12^. The influence of pressing the masticated chewing gums to a 1 mm thick wafer has been addressed in previous studies^12,13,17,24^; although there are no studies that focus solely on this topic, the overall results of previous studies suggest that accurate mixture information can be extracted from a 1 mm thick wafer if the pressing procedure was conducted using a calibrated press. Fourthly, the flattened wafer was scanned on both sides using a Canoscan Lide 220® flatbed scanner (300 dpi, standard calibration parameters for colour digitalization). Fifthly, the digital images of the samples were saved in uncompressed TIFF format.

The MP of each subject was measured on three occasions, with an interval of two days between sessions. At the end of the first and second sessions subjects were provided with a sealed box containing 15 pieces of the test-food chewing-gum. The subjects were instructed to chew on two of these new gums at the same time for one minute, one hour after meals until the next session.

### Digital image analysis

The mixture quantification procedure employed a commonly used segmentation → feature extraction → MP assessment approach^12,13,16^. The digital images obtained from the flattened chewing-gum samples were segmented to isolate the area of the bolus against the background using a fully-automated colour-based segmentation algorithm, constructed upon the combination of Mean Shift^25,26^, Distance Map, and K-Means classification algorithms^27^. This segmentation algorithm was implemented in a Python 3 script following the instructions provided by Vaccaro (2018):

1. Stablish the mean background colour in the CIE Lab colour space (*bg*) as the mean colour of the super-pixels (clusters) resultant from the Mean Shift segmentation positioned in the four corners of the image.
2. For each pixel *x*_*i*_ of the segmented image, compute the logarithm of the colour Euclidean distance (*d*_*i*_) to *bg* (*d*_*i*_ = log ||*x*_*i*_ − *b*||) and form a new Distance Map (DM) image to decrease the heterogenicity between regions of the bolus and preserves the homogeneity of the background.
3. Classify the pixels in the DM in two clusters using the K-Means procedure (*k* = 2).
4. Select the position of the pixels in the cluster located in the centre of the image as the bolus.
5. Extract the pixel values corresponding to the bolus in the original image.

Afterwards, the MP of the sample was analysed by computing the Variance of the Histogram of the Hue channel of the HSI colour space (VhH). The VhH has been used previously as a proxy measure of the mixture of two-coloured chewing-gums^12,17^.

### Statistical analysis

Statistical analyses were performed in IBM® SPSS® Statistics 25. The entire image batch was processed twice consecutively to evaluate the consistency of the VhH computation, and the systematic error was assessed by a paired t-test. The normality of MP measures per session grouped by the chewing-gum consumption rate was verified by both Kolmogorov-Smirnov and Shapiro-Wilk tests. The association between the age (continuous IV) and the chewing gum consumption rate (categorical DV) was measured with the eta-squared statistic (*η*^2^); furthermore, this analysis was used to estimate the effect size of the age on the chewing-gum consumption rate.

Each participant was exposed to same conditions for the same number of times during the experiment. The differences in MP were assumed to be due the chewing-gums consumption rate (CGC), the prescribed increase in chewing-gum consumption per session (SESSION), and from error or unexplained variation (ERROR). The MP of individuals that consumed less than one (CGC = 0), less than 4 (CGC = 1), and 4 or more chewing-gums per month (CGC = 2) was measured three times: before prescription (SESSION = 1), two days after being prescribed with an increase of chewing-gum consumption (SESSION = 2), and two days after the las session while continuing the same prescription (SESSION = 3).

If the regular consumption of chewing-gums increases the chances of achieving a greater mixture degree on two-coloured chewing gum mixing tests; then, we expect the MP to improve after the subjects are prescribed to increase in the chewing-gum consuming rate, especially for non-regular consumers. Therefore, a 3 × 3 mixed ANOVA with SESSION as the within-subject factor and CGC as the between-subjects factor was run. Sphericity of the variances was verified using the Mauchly’s test of Sphericity^28^. In case that the Sphericity was violated, then, the Greenhouse-Geisser or the Huynh-Feldt corrections would be used^29,30^. Session-wise and group-wise comparison were performed with post hoc Bonferroni.

Finally, the effects of the sex, the CGC, the Dental Status, the age and the SESSION stage over the MP were evaluated using Generalized Estimating Equations (GEE). The GEE model consisted of a normal probability distribution with identity link function, with SESSION as the within-subject effect and 3 measurements per subject.

## Results

The complete dataset is included in Supplementary Table S1. Descriptive demographic data for age groups, dental status, chewing gum consumption and sex is detailed in Table 1. Additionally, the descriptive information about distribution of subjects between AG clusters is detailed in Table 2. A total of 795 flattened chewing-gums accounted for 1590 digital images (one image per side). On average, the time required to perform the clinical procedure and retrieve a single sample was 2 minutes and 46 seconds. Then, the image processing step required a total of 3 hours and 11 minutes on a desktop computer with an Intel® Core™ i7 7700 K with 32GB of RAM. No systematic error was detected for VhH computation applied over the same batch of images (*p* = 1.00).

**Table 1:** Descriptive demographic data for age groups, dental status, chewing gum consumption and sex.

Age group	Dental status	Chewing gums consumption per month	Sex	Frequency	Percent	Cumulative Percent
Between 15 and 24 years old	Natural	Less than one piece	Male	3	42.9	42.9
			Female	4	57.1	100.0
			Total	7	100.0	
		Between one and four pieces	Male	13	41.9	41.9
			Female	18	58.1	100.0
			Total	31	100.0	
		More than four pieces	Male	13	39.4	39.4
			Female	20	60.6	100.0
			Total	33	100.0	
Between 25 and 44 years old	Natural	Less than one piece	Male	15	60.0	60.0
			Female	10	40.0	100.0
			Total	25	100.0	
		Between one and four pieces	Male	3	27.3	27.3
			Female	8	72.7	100.0
			Total	11	100.0	
		More than four pieces	Male	8	61.5	61.5
			Female	5	38.5	100.0
			Total	13	100.0	
	Artificial denture	Less than one piece	Male	2	66.7	66.7
			Female	1	33.3	100.0
			Total	3	100.0	
		Between one and four pieces	Female	1	100.0	100.0
Between 45 and 65 years old	Natural	Less than one piece	Male	21	53.8	53.8
			Female	18	46.2	100.0
			Total	39	100.0	
		Between one and four pieces	Male	3	75.0	75.0
			Female	1	25.0	100.0
			Total	4	100.0	
		More than four pieces	Male	3	75.0	75.0
			Female	1	25.0	100.0
			Total	4	100.0	
	Artificial denture	Less than one piece	Male	7	38.9	38.9
			Female	11	61.1	100.0
			Total	18	100.0	
		Between one and four pieces	Male	1	20.0	20.0
			Female	4	80.0	100.0
			Total	5	100.0	
More than 65 years old	Natural	Less than one piece	Male	20	51.3	51.3
			Female	19	48.7	100.0
			Total	39	100.0	
		Between one and four pieces	Female	1	100.0	100.0
	Artificial denture	Less than one piece	Male	12	38.7	38.7
			Female	19	61.3	100.0
			Total	31	100.0

**Table 2:** Descriptive statistics of the age of subjects per age groups (AG).

AG	Description	*N*	Min (years)	Max (years)	Mean(years)	Standard Dev. (years)
1	Less than 25 years old	71	18	24	20.916	0.243
2	Between 25 and 44 years old	53	25	44	32.962	0.964
3	Between 45 and 64 years old	70	45	64	54.500	0.688
4	More than 64 years old	71	65	90	76.507	0.887

**Figure 1:**
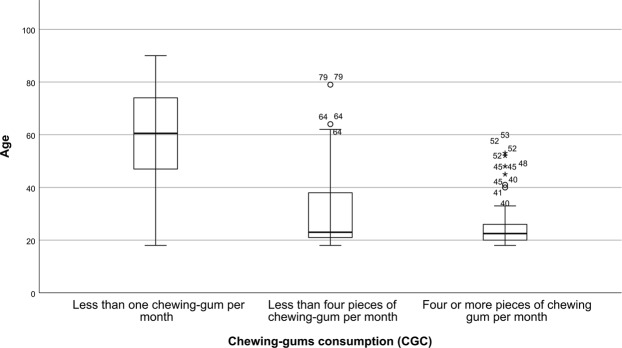
Box plots of the age of the subjects grouped by the chewing-gums consumption rate (CGC).

There was a high association between the age and the chewing-gum consumption rate, with *η*^2^ = 0.572. This suggest that the age can explain, at least partly, the amount of chewing-gums that patients consume. A visual representation of the relationships between the AG and the CGC is show on Fig. 1. The Kolmogorov-Smirnov and Shapiro-Wilk tests confirmed the normality of the distribution of MP measures; with *p* > 0.143 when grouping by CGC, Dental Status and Sex. On the other hand, the assumption normality was rejected for non-grouped MP measurements with p < 0.001. The violation of the assumption of normality for non-grouped MP measurements was expected due the known differences in the masticatory capabilities between subjects of different ages. Table 3 provides descriptive statistics about the age and MP distributions between SESSION and GCG groups.

**Table 3:** Descriptive statistics about the masticatory performance and age distributions between sessions (SESSION = 1, 2 and 3) and chewing-gum consumption rates for less than one, less than 4, and 4 or more chewing-gums per month (CGC = 0, 1, 2 respectively).

CGC	SESSION	Masticatory performance (VhH)				*N*	Age (years)			
		Min	Max	Mean	Std. Dev.		Min	Max	Mean	Std. Dev.
0	1	1.46e + 07	2.22e + 07	1.80e + 07	1.44e + 06	162	18	90	59.41	18.42
	2	1.49e + 07	2.51e + 07	1.94e + 07	2.08e + 06					
	3	1.47e + 07	2.50e + 07	1.95e + 07	2.22e + 06					
1	1	1.80e + 07	2.42e + 07	2.14e + 07	1.53e + 06	53	18	79	30.23	14.62
	2	1.92e + 07	2.79e + 07	2.25e + 07	1.91e + 06					
	3	1.92e + 07	2.57e + 07	2.27e + 07	1.74e + 06					
2	1	1.97e + 07	2.61e + 07	2.35e + 07	1.29e + 06	50	18	53	25.04	8.86
	2	2.06e + 07	2.77e + 07	2.35e + 07	1.35e + 06					
	3	2.08e + 07	2.58e + 07	2.35e + 07	1.12e + 06					

The Masticatory Performance was measured using the Variance of the Histogram of the Hue (VhH).

The main effect of SESSION violates the sphericity assumption in Mauchly’s sphericity test for the repeated measures variable; with Mauchly’z W = 0.839, approximated χ^2^ = 45.960, df = 2 and *p* < *0.001*. Therefore, the *F*-value for the main effect of SESSION and its interaction with the between-group variable CGC needed to be corrected for violations of sphericity. Given that the estimated ε is greater than 0.75; then, the Huynh-Feldt correction was used.

The Table 4 provides a summary of the repeated measures effects in the ANOVA with corrected F-values. There was a significant main effect of SESSION, *F*(1.746, 457.328) = 59.075, p < 0.001, *η*_p_^2^ = 0.184. Furthermore, the pairwise comparisons using the Bonferroni adjustment for the main effect of SESSION are provided in Table 5. There were significant differences between SESSION = 1 and SESSION = 2 (before and after 2 days of increased chewing-gum consumption), and between SESSION = 1 and SESSION = 3 (before and after 4 days of increased chewing-gum consumption), with *p* = 0.001; but there were no differences between SESSION = 2 and SESSION = 3 (after 2 days and after 4 days of increased chewing-gum consumption), with *p* = 0.700.

**Table 4:** Summary of the within-subject effects with corrected *F*-values for masticatory performance measures, with α = 0.05.

Source	Type III Sum of Squares		df	Mean Square	*F*	Sig.	*η*_p_^2^
SESSION	Huynh-Feldt	1.08e + 14	1.746	6.17e + 13	59.075	0.000	0.184
	Lower-bound	1.08e + 14	1.000	1.08e + 14	59.075	0.000	0.184
SESSION * CGC	Huynh-Feldt	5.55e + 13	3.491	1.59e + 13	15.225	0.000	0.104
	Lower-bound	5.55e + 13	2.000	2.77e + 13	15.225	0.000	0.104
Error (SESSION)	Huynh-Feldt	4.77e + 14	457.328	1.04e + 12			
	Lower-bound	4.77e + 14	262.000	1.82e + 12			

Where prescribed increase in chewing-gums consumption (SESSION) is the within-subject factor and the chewing-gums consumption rate (CGC) is the between-subjects factor.

**Table 5:** Pairwise comparisons using the Bonferroni adjustment for the main effect of the prescribed increase in chewing-gums consumption (SESSION) over the Masticatory Performance, with α = 0.05.

(I) SESSION	(J) SESSION	Mean Difference (I-J)	Std. Error	Sig.	95% Confidence Interval for Difference	
					Lower Bound	Upper Bound
1	2	−851081.65	100609.62	0.000	−1093495.56	−608667.75
	3	−940172.37	108029.16	0.000	−1200463.29	−679881.46
2	1	851081.65	100609.62	0.000	608667.75	1093495.56
	3	−89090.72	74603.81	0.700	−268844.91	90663.47
3	1	940172.37	108029.16	0.000	679881.46	1200463.29
	2	89090.72	74603.81	0.700	−90663.47	268844.91

However, Levene’s test indicates that variances are not homogeneous for all levels of the repeated measured variables (p < 0.001). Therefore, an additional one-way ANOVA using the Welch F test and the Games-Howell correction was performed, considering CGC as the fixed factor. This secondary test confirmed that there was a significant effect of CGC, with *F* (2, 356.53) = 564.73, *p* < *0.001*. Furthermore, pairwise comparison tests using Games-Howell provided in Table 6, showed that there are significant differences in MP measures between CGC = 0 and CGC = 1 (less than one chewing-gum and less than 4 chewing-gums per month), between CGC = 1 and CGC = 2 (less than four chewing-gum and 4 or more chewing-gums per month), and between CGC = 0 and CGC = 2 (less than one chewing-gum and 4 or more chewing-gums per month).

**Table 6:** Pairwise comparisons using the Games-Howell adjustment for the main effect of chewing-gum consumption rates: less than one, less than 4, and 4 or more chewing-gums per month (CGC = 0, 1, 2 respectively); over the Masticatory Performance, with α = 0.05.

(I) CGC	(J) CGC	Mean Difference (I-J)	Std. Error	Sig.	95% Confidence Interval for Difference	
					Lower Bound	Upper Bound
0	1	−3.22e + 06	171449.35	0.000	−3.63e + 06	−2.82e + 06
	2	−4.54e + 06	138268.80	0.000	−4.87e + 06	−4.22e + 06
1	2	3.22e + 06	171449.35	0.000	2.82e + 06	3.63e + 06
	3	−1.32e + 06	176197.40	0.000	−1.73e + 06	−9.05e + 05
2	0	4.54e + 06	138268.80	0.000	4.22e + 06	4.87e + 06
	3	1.32e + 06	176197.40	0.000	9.05e + 05	1.73e + 06

**Figure 2:**
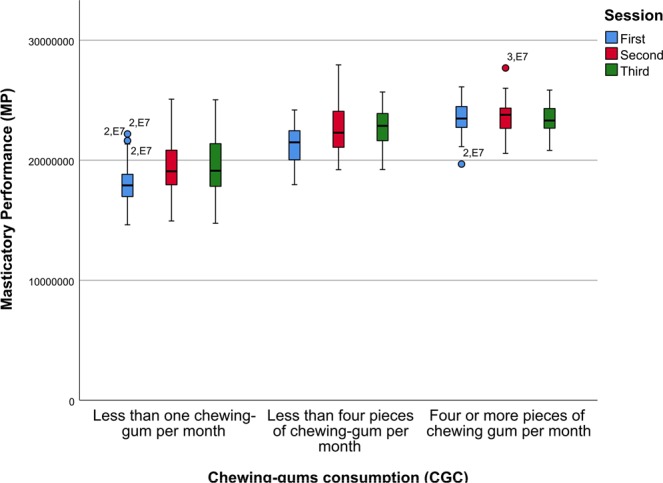
Box plots of Masticatory Performance (MP) measures grouped by the chewing-gums consumption rate (CGC) and SESSION to graphically represent the effects of CGC on MP.

Furthermore, the GEE results detailed on Table 7 confirm the influence of the prescribed increase in the consumption of chewing gums, where both the second and third sessions reported a significant effect over the MP regression model (B = 3.18e + 06, Wald Chi-Square = 91.063, p < 0.001 and B = 3.20e + 06, Wald Chi-Square = 86.02, p < 0.001 respectively). Also, the results of the GEE confirm the influence of the CGC over the MP regression model; where a medium chewing gum consumption (CGC = 1) and high chewing gum consumption (CGC = 2) showed a significant effect over the MP (B = 1.98e + 06, Wald Chi-Square = 16.505, p < 0.001 and B = 3.21e + 06, Wald Chi-Square = 34.706, p < 0.001). In this regard, the Fig. 2 shows box-plots of MP measures grouped by CGC and SESSION to graphically represent the effects of different rates of chewing-gum consumption on MP; furthermore, this plot also helps to visualize the effects of prescribing a controlled increase in the consumption of chewing-gums.

**Table 7:** Parameter estimates of the Generalized Estimating Equations model.

Parameter	B	Std. Error	95% Wald Confidence Interval		Hypothesis Test		
			Lower	Upper	Wald Chi-Square	df	Sig.
Intercept	2.08e + 07	3.98e + 05	2.00e + 07	2.16e + 07	2737.763	1	0.000
Sex	2.40e + 05	5.53e + 05	−8.44e + 05	1.32e + 06	0.188	1	0.664
CGC = 2	3.21e + 06	5.44e + 05	2.14e + 06	4.27e + 06	34.706	1	0.000
CGC = 1	1.98e + 06	4.87e + 05	1.03e + 06	2.94e + 06	16.505	1	0.000
Dental Status	−1.49e + 06	8.25e + 05	−3.11e + 06	1.28e + 05	3.255	1	0.071
Session = 3	3.20e + 06	3.45e + 05	2.53e + 06	3.88e + 06	86.020	1	0.000
Session = 2	3.18e + 06	3.34e + 05	2.53e + 06	3.84e + 06	91.063	1	0.000
Age	−4.51e + 04	6.48e + 03	−5.78e + 04	−3.24e + 04	48.406	1	0.000
Sex * [CGC = 2]	−3.27e + 05	4.58e + 05	−1.22e + 06	5.72e + 05	0.508	1	0.476
Sex * [CGC = 1]	−5.56e + 05	4.51e + 05	−1.44e + 06	3.27e + 05	1.520	1	0.218
Sex * Dental Status	−3.65e + 05	3.79e + 05	−1.11e + 06	3.78e + 05	0.927	1	0.336
Sex * Age	−6.41e + 03	9.04e + 03	−2.41e + 04	1.13e + 04	0.503	1	0.478
[CGC = 1] * Dental Status	−2.09e + 04	4.71e + 05	−9.44e + 05	9.02e + 05	0.002	1	0.965
[CGC = 2] * [Session = 3]	−2.62e + 06	2.96e + 05	−3.20e + 06	−2.04e + 06	78.199	1	0.000
[CGC = 2] * [Session = 2]	−2.52e + 06	2.57e + 05	−3.02e + 06	−2.01e + 06	95.665	1	0.000
[CGC = 1] * [Session = 3]	−1.13e + 06	2.50e + 05	−1.62e + 06	−6.44e + 05	20.535	1	0.000
[CGC = 1] * [Session = 2]	−1.22e + 06	2.58e + 05	−1.73e + 06	−7.17e + 05	22.478	1	0.000
[CGC = 2] * Age	2.94e + 04	1.46e + 04	8.08e + 02	5.80e + 04	4.062	1	0.044
[CGC = 1] * Age	1.20e + 04	1.15e + 04	−1.05e + 04	3.46e + 04	1.096	1	0.295
Dental Status * [Session = 3]	−9.66e + 05	2.40e + 05	−1.44e + 06	−4.96e + 05	16.221	1	0.000
Dental Status * [Session = 2]	−6.50e + 05	2.25e + 05	−1.09e + 06	−2.08e + 05	8.330	1	0.004
Dental Status * Age	2.15e + 04	1.20e + 04	−2.06e + 03	4.50e + 04	3.199	1	0.074
[Session = 3] * Age	−2.29e + 04	5.87e + 03	−3.44e + 04	−1.14e + 04	15.175	1	0.000
[Session = 2] * Age	−2.60e + 04	5.50e + 03	−3.67e + 04	−1.52e + 04	22.242	1	0.000
(Scale)	1.84e + 12

On the other hand, the GEE results show that the age of the patient had a significant inverse effect over the MP (B = −4.51e + 04, Wald Chi-Square = 48.406, p < 0.001); this indicates that the MP decreases with the age. Also, the interaction of a high chewing gum consumption and the age of the patient ([CGC = 2] * Age) proved to have a significant effect over the MP (B = 2.94e + 04, Wald Chi-Square = 4.062, p = 0.044); however, the interaction of a medium chewing gum consumption and the age of the patient ([CGC = 1] * Age) did not show a significant effect over the MP (B = 1.20e + 04, Wald Chi-Square = 1.096, p = 0.295). Furthermore, the dental status did not show a significant effect over the MP by itself (B = −1.49e + 06, Wald Chi-Square = 3.255, p = 0.071); but the interaction between the dental status and the second and third sessions (Dental Status * [Session = 2] and Dental Status * [Session = 3]) exhibited significant inverse effects over MP (B = −6.50e + 05, Wald Chi-Square = 8.330, p = 0.004 and B = −9.66e + 05, Wald Chi-Square = 16.221, p < 0.001).

## Discussions

Within the limitations of this experiment, results suggest that there is an association between the age and the rate of consumption of chewing-gums (*η*^2^ = 0.572); thus, rejecting the first null hypothesis. This was an expected outcome, as previous empirical examination indicated that most of the patients over 60 years old reported not to consume chewing-gums at all; while patients younger than 30 years old consumed chewing-gums in a regular fashion. Nonetheless, it was not within the scope of this study to determine the reasons behind this phenomenon. On the other hand; these results suggest that elderly people, on average, are not used to chew on chewing-gums. Therefore, the outcome of the two-coloured chewing gum mixing test on elderly people might be influenced by the obstacles of dealing with a strange type of food.

On the other hand, the GEE results suggest that the interaction between consuming less than four chewing gums per month and the age ([CGC = 1] * Age) did not had a significant effect over the MP; but the interaction of consuming more than four chewing gums per month and the age ([CGC = 2] * Age) proved to produce an effect over the MP. The lack of effect of the interaction [CGC = 1] * Age over the MP can be explained by the significant increase in the MP measurements of the CGC = 1 between sessions. Furthermore, the results of this study suggest that there are significant differences between the MP of people that consumed less than 1, less than four, and four-or-more chewing-gums per month; thus, rejecting the second null hypothesis. Again, this phenomenon could be explained by the transitivity of already known relationships: consumption of chewing-gums *is related to* age & age *is related to* MP.

However, one of the objectives of this study was to determine if a low MP related to a low consumption of chewing-gums could be improved solely by prescribing a controlled increase in the consumption of chewing-gums. In this regard, the results of this study suggest that there was a significant effect of the prescription on the MP with *F*(1.746, 457.328) = 59.075, p < 0.001, *η*_p_^2^ = 0.184; thus rejecting the third null hypothesis. Moreover, results suggest that the improvement in the MP produced by the prescription was focused right after the first session, as there were no differences in MP measures between SESSION 2 and 3.

In the light of above-mentioned results, we consider important to suggest that, whenever possible, instruct the patient to chew on a few chewing-gums before taking the mixing-test. However, the limitations of the present study prevent us for stablishing the quantity and the frequency of consumption of extra chewing-gums needed to achieve the observed stability of MP measures.

Between the limitations of this study, it is important to notice that the size and variances in the MP measures of the CGC groups was uneven. Also, the sampling procedure was not fully randomized, as there was a limited number of subjects that passed the inclusion and exclusion criteria within the available population. Therefore, we consider that further research in this area should pursue a larger and more evenly distributed study sample; moreover, this study should be extended to use other combinations of chewing-gums brands and colours available in other countries and regions.

## Conclusion

This study found evidence that sustains the hypothesis that the regular consumption of chewing gums is inversely related to the age of the patient. Furthermore, there is evidence that the two-coloured chewing gum mixing test for MP assessment can be influenced by the chewing-gum consumption habits of the patient, and that this issue can be overcome by prescribing the patient with a controlled increase in the consumption of chewing-gums for a few days.

## Supplementary information

Supplementary Table S1

## Data Availability

The datasets generated and/or analysed in this study are included in this published article (and its Supplementary Information files).
